# Prevalence, risk factor and outcome in middle-aged and elderly population affected by hemiplegic shoulder pain: An observational study

**DOI:** 10.3389/fneur.2022.1041263

**Published:** 2023-01-12

**Authors:** Yaomei Li, Siqi Yang, Lijun Cui, Yong Bao, Lin Gu, Huijuan Pan, Jixian Wang, Qing Xie

**Affiliations:** ^1^Department of Rehabilitation Medicine, Ruijin Hospital, Shanghai Jiao Tong University School of Medicine, Shanghai, China; ^2^Department of Rehabilitation Medicine, Shanghai Ruijin Rehabilitation Hospital, Shanghai, China

**Keywords:** hemiplegic shoulder pain, stroke, rehabilitation, risk factors, functional outcomes

## Abstract

**Background:**

Hemiplegic shoulder pain is the most common complication after stroke. It usually occurs during the critical period of stroke recovery and hinders the rehabilitation of upper extremity motor function. However, there are few studies on the risk factors, the development and prognosis of shoulder pain after stroke.

**Objectives:**

This study aimed to observe the prevalence of post-stroke shoulder pain in the middle-aged and elderly population, find out the risk factors for post-stroke shoulder pain, and explore its effect on stroke outcome.

**Methods:**

Eligible patients with hemiplegic shoulder pain in the rehabilitation unit were recruited and followed up at 2 and 4 months. The basic clinical information including age, gender, hypertension and atrial fibrillation history, stroke types, stroke location was recorded. Range of motion for shoulder, glenohumeral subluxation, muscle tension, activity of daily living of upper limb were measured. Data from blood test and shoulder ultrasonography were collected.

**Results:**

480 stroke patients were screened within 1 year, and 239 patients were included in the statistical analysis. The prevalence of hemiplegic shoulder pain was 55.6% (133/239) at admission, 59.4% (142/239) after 2 months, and 55.1% (130/236) after 4 months. We found that shoulder pain was more likely to occur in women, patients with large-area stroke, increased tension of biceps brachii or triceps brachii, subluxation and limited passive range of motion of the shoulder. And the ability of daily living of patients with shoulder pain was significantly lower than that of patients without shoulder pain. Shoulder ultrasonography showed that the most common lesion in patients with shoulder pain was supraspinatus tendon thickening, and the thickness of supraspinatus tendon in the hemiplegic side of patients with shoulder pain was significantly higher than that of unaffected side. In addition, the hospitalization rate of patients with shoulder pain after 2 months and 4 months was significantly higher than that without shoulder pain.

**Conclusions:**

Hemiplegic shoulder pain has a high prevalence and can last for several months. Multiple risk factors are involved. Moreover, hemiplegic shoulder pain affects the readmission rate of patients. Therefore, we should pay more attention to this problem in our clinical work. The application of various means to relieve shoulder pain will be conducive to the recovery of upper limb motor function and shorten the in-hospital rehabilitation time.

## Introduction

Hemiplegic shoulder pain (HSP) is the most common complication of stroke patients and has a high incidence rate of 24–64% in the inpatient rehabilitation unit (1). Shoulder pain usually occurs within 2–3 months after stroke (2), which is a critical period of upper limb recovery. Studies have shown that hemiplegic shoulder pain prolongs the hospitalization time of patients and aggravates the medical burden (3). Among patients with shoulder pain, moderate-severe pain was predominant (4). A study (5) found that the prevalence of shoulder pain at 12 months after stroke is similar to that at 4 months after stroke. Long-term pain not only delays the recovery of upper limb function, but also leads to sleep disorders, anxiety, depression, and other psychological problems (6), which lead to decreased quality of life of patients. Therefore, finding out the risk factors of HSP has positive clinical significance and can help to achieve early prevention, and individualized treatment and reduce the incidence of HSP.

HSP is generally considered to be the result of multiple factors, including mechanical and neurological factors. Mechanical factors include gleno-humeral subluxation (7), rotator cuff injury (8), biceps tendinitis, glenohumeral joint disease, adhesive arthritis, direct trauma, etc. Neurological factors include soft paralysis (9), spasm, brachial plexus injury, complex regional pain syndrome (CRPS) (10), central sensitization (11), paresthesia (12), unilateral neglect, etc. Recent studies have found that other factors, such as diabetes mellitus (13), vitamin D3 deficiency (14), psychology (15) and immunity (16), may also be associated with HSP.

Many treatments can be used to relieve shoulder pain after stroke. Traditional treatment methods include pills, electrical stimulation (17), kinesio taping (18), arm orthosis (19) to protect shoulder joint, closed injection to suppress immune response, shoulder training and other exercise therapies to promote functional recovery of upper limbs and shoulders, traditional Chinese medicine fumigation and acupuncture (20). Although most patients benefit from traditional treatment, the remaining the patients with persistent shoulder pain do not get relief after using traditional treatment. Recent years, emerging treatments such as botox injection (21), suprascapular nerve block (22), shockwave therapy (23), platelet-rich plasma (PRP) injection (24) and robot-assisted therapy (25) are being used to treat HSP.

Previous studies of HSP have mostly been retrospective, with a large span of young age, and a lack of objective data (4, 13, 26). Compared with elderly patients, young stroke patients rarely suffer from hypertension and diabetes, which are the important possible risk factors of HSP. In this study, we focused on the middle-aged and elderly population and prospectively observed the objective factors associated with HSP. We aimed to observe the prevalence of post-stroke shoulder pain, identify the risk factors for HSP by comparing basic clinical information, laboratory tests, and clinical examinations in stroke patients with or without shoulder pain, and investigate their impact on stroke outcomes.

## Methods

### Subjects

**Figure 1:**
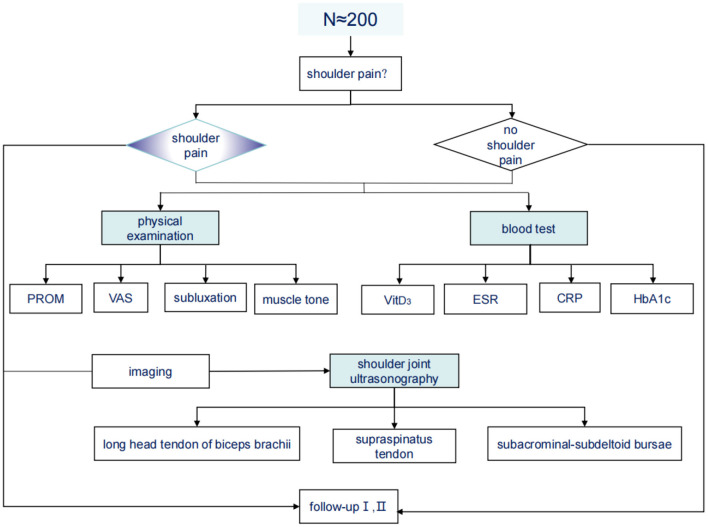
Study flow chart.

From October 1, 2020 to October 31, 2021, any patient hospitalized in the Ruijin rehabilitation ward with stroke was screened. The first evaluation was performed within 1 week after admission, and the evaluation mainly included shoulder pain questionnaire and relevant clinical physical examination (see Appendix for details). Routine blood test data and shoulder ultrasound findings were recorded (Figure 1). After 2 and 4 months of systematic rehabilitation treatment, the patients were followed up and evaluated to observe the changes of shoulder pain. The inclusion criteria were as follows: Meet the diagnostic criteria of cerebral infarction or intracerebral hemorrhage and confirmed by brain computerized tomography (CT) scan or magnetic resonance imaging (MR). Stroke was diagnosed according to the WHO definition (27) as rapidly developing signs of focal (or global) disturbance of cerebral function lasting more than 24 h with no obvious cause except vascular origin; First stroke attack; Duration of stroke <12 months; Age>50 years.; Stay in the hospital >7 days. The exclusion criteria were as follows: Serious consciousness, cognitive and mental disorders; Patients with severe medical diseases and unstable vital signs; Hemiplegia after intracranial aneurysm embolization; Patients who refuse to be examined (4, 28, 29).

### Assessment methods

After screening, we conducted a shoulder pain questionnaire survey, which mainly included: whether there was shoulder pain; Visual Analog Scale (VAS) score of shoulder pain; site of shoulder pain, whether shoulder pain occurred before stroke (if occurred, whether shoulder pain worsened after stroke); time of pain initiation, frequency of pain; the relationship between shoulder pain and movement; whether shoulder pain affected sleep (30, 31).

We collected the following basic clinical information, including: age, gender, history of hypertension and atrial fibrillation, time of onset, type of stroke (cerebral infarction, intracerebral hemorrhage, subarachnoid hemorrhage), hemiplegic side, stroke area and location (large-area, cortex, brainstem, basal ganglia, thalamus, pons, undefined). “Undefined” refers to specific stroke location not identified due to lack of examination and reporting.

We conducted clinical physical examination to measure the passive range of motion of shoulder joint (PROM, including flexion, abduction, internal rotation and external rotation) (32), palpation of gleno-humeral subluxation (4), soft tissue shoulder palpation (biceps tendon, pectoralis major muscle, acromion glide sac, infracoracoid process, teres major, teres minor, etc.) (26), and evaluate of muscle tone of biceps brachii and triceps brachii. We defined restricted shoulder PROM as three degrees: flexion/abduction 140°-180°or internal/external rotation 60°-90°as mild restricted; flexion/abduction 90°-140°or internal/external rotation 45°-60°as moderate restricted; flexion/abduction 0°-90°or internal/external rotation 0°-45°as severe restricted. During the shoulder examination, the patient was asked whether pain occurred and to grade a VAS score of shoulder pain. Disability has a profound effect on HRQoL in stroke. Disability measures include modified Rankin Scale, Barthel Index, AHA Stroke Outcome Scale, and the Stroke Levity Scale (33). And we assessed disability by Modified Barthel Index (MBI) form regarding upper limbs, which sum up to 30 points, including feeding, bathing, dressing, and grooming (12).

Considering shoulder pain may be associated with infection, immune (16), diabetes mellitus (34) and bone metabolism (14, 35), we collected related routine blood examination data, including leukocyte count, percentage of neutrophils, C-reactive protein (CRP), serum amyloid protein A (SAA), erythrocyte sedimentation rate (ESR), Vitamin D3, glycosylated hemoglobin (HbA1c), fasting plasma glucose (FPG) and 2-h postprandial plasma glucose (P2hPG) (28) to test their relationship with shoulder pain. Some patients underwent shoulder joint ultrasonography during hospitalization to assess the long head of biceps tendon, supraspinatus tendon, and subacrominal-subdeltoid (SA-SD) bursae of both shoulders, which helped to analyze the cause of shoulder pain. In our observational research, it is a pity that not all patients have undergone shoulder ultrasonography, which may lead to bias.

Patients with HSP were followed up and evaluated at 2 and 4 months of systematic rehabilitation. For readmitted patients, the follow-up evaluation content was the same as the first. While shoulder pain questionnaires were administered to inpatients elsewhere, outpatients and home-based rehabilitation patients. We have dedicated evaluators to do the assessments and ultrasonography were conducted by ultrasound doctor, and all of them were not informed of patients' grouping.

### Statistical analysis

The data were statistically analyzed by IBM SPSS statistics 24 software. Using chi square test (13), we examined if there was an association between shoulder pain and gender, hypertension and atrial fibrillation history, main type of stroke, hemiplegic side, respectively, stroke location such as massive, cortical, brain stem, basal gangregion, thalamic and pontine, respectively, the incidence of dystonia (Biceps brachii or triceps brachii, increased or decreased, respectively) and subluxation, the degree of shoulder PROM, the rate of increased CRP, SAA and ESR, and the rate of decreased Vitamin D3.

Using *t*-test (4), we examined if there was an association between shoulder pain and age, activities of daily living involving feeding, bathing, dressing, and grooming, leukocyte count, percentage of neutrophils, CRP, ESR, HbA1c, FPG, P2hPG, Vitamin D3, and we also examined the difference of supraspinatus tendon thickness between hemiplegic side and healthy side in patients with shoulder pain.

Probability values of <0.05 were considered significant.

When testing whether there is a difference in the ratio of bilateral hemiplegia between patients with shoulder pain and patients without shoulder pain, the minimum expected count is 1.33, so we used the continuous modified chi square test. When testing whether there is a difference in the rate of thalamic stroke between patients with shoulder pain and patients without shoulder pain, the minimum expected count was 3.99, so we used the continuous modified chi square test. For shoulder PROM, we divided it into three levels: mild limitation: forward flexion/abduction 140°-180°, or internal/external rotation 60°-90°; moderate limitation: flexion/abduction 90°-140°, or internal/external rotation 45°-60°; severe limitation: flexion/abduction 0°-90°, or internal/external rotation 0°-45°. For vitamin D3 deficiency, we also divided it into three levels: mild: vitamin D3 (40, 50 nmol /L); moderate: vitamin D3 (20, 40 nmol/L); severe: vitamin D3 <20 nmol/L, and tested their relationship with shoulder pain, respectively. When we used R^*^C chi square test to test whether there were differences in stroke types between patients with shoulder pain and patients without shoulder pain, there were two expected values <5 and the minimum expected count was 1.33, so we chose likelihood ratio test.

## Results

**Figure 2:**
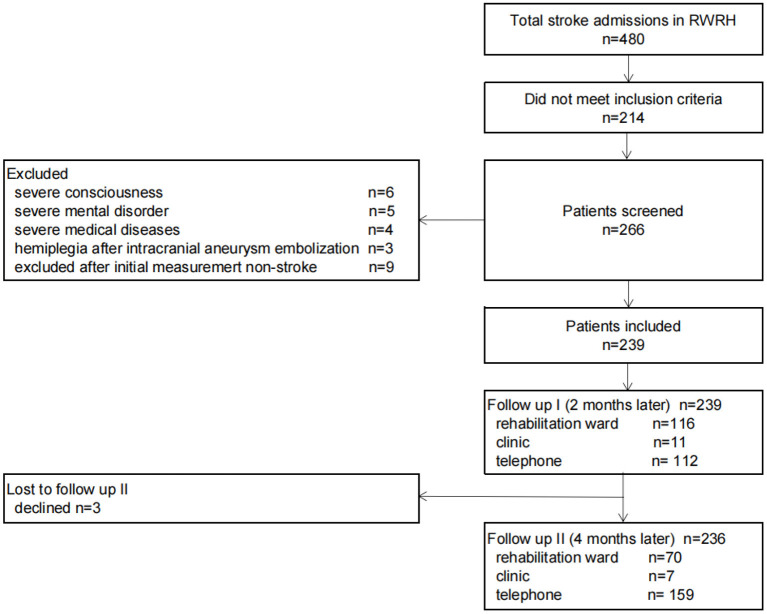
Patient recruitment flowchart.

From October 1, 2020 to October 31, 2021, all patients admitted to the Rehabilitation ward of Ruijin Hospital diagnosed with stroke underwent preliminary screening (Figure 2). A total of 480 stroke patients were screened. According to the inclusion and exclusion criteria, 6 patients with severe mental disorders, 5 patients with severe mental disorders, 4 patients with severe medical diseases, 3 patients with hemiplegia after intracranial aneurysm embolization and 9 patients with stroke symptoms but diagnosed with other neurological diseases were excluded. Therefore, 239 patients were finally evaluated for the first time. The first follow-up was conducted after 2 months. One hundred and sixteen readmitted patients were measured again, 11 patients were assessed in clinic and 112 patients were followed by phone. The second follow-up was conducted after 4 months. A total of 236 survivors were followed up (3 patients died, and the causes of death were esophageal malignant tumor, pancreatic cancer and recurrent large-area cerebral infarction, respectively). Among them, 70 patients were re-hospitalized and completed the follow-up in the rehabilitation ward, 7 patients were assessed in clinic and 159 patients were followed by telephone.

### The prevalence of HSP

**Figure 3:**
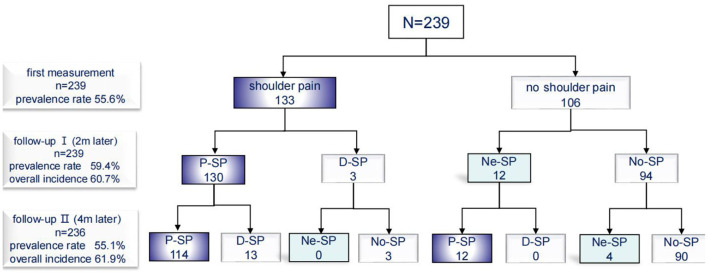
Incidence of shoulder pain. P-SP, persisted shoulder pain; D-SP, dissolved shoulder pain; Ne-SP, new developed shoulder pain; No-SP, no shoulder pain.

Initial assessment was performed within seven days of admission, and 133 (56%) of the 239 suffered from HSP. Most patients suffered shoulder pain after stroke. Ten patients reported a history of shoulder pain before stroke, and 8 of them worsened after stroke. Seventy eight (64%) of the 230 patients reported pain herself/himself, and other patients were detected by caregivers or assessors. One hundred and four (85%) shoulder pain occurred after shoulder movements or exercises, and 50 (30%) shoulder pain occurred at rest. At follow-up I, 59% (133-3+12)/239) patients suffered shoulder pain (3 shoulder pain relieved, 130 shoulder pain persisted and 12 newly developed shoulder pain). At follow-up II, 55% (133-3-13+12+4)/236) patients suffered shoulder pain (13 shoulder pain relieved, 126 shoulder pain persisted and 4 newly developed shoulder pain) (Figure 3).

### Relationship between HSP and basic clinical factors

Our study reported 239 patients who had initial and two follow-ups data, with an average age of 70 ± 9 years old, ranged from 50 to 94 years, 148 (62%) male, 197 (82%) infarcts, 115 (48%) left hemiplegic, 201 (84%) comorbidity with hypertension, 31 (13%) comorbidity with atrial fibrillation, 27 (11%) large-area cerebral infarction, 38 (16%) cortical cerebral infarction, 34 (14%) brain stem infarction, including 20 (8%) pontine infarction, 35 (15%)basal ganglia infarction, 9 (4%) thalamic infarction, 8 (3%) undefined (Table 1).

**Table 1:** Basic clinical factors and their relation to HSP at initial assessment and follow-ups.

	**Initial assessment**				**Follow up I**				**Follow up II**			
	**Total (*****n** =* **239)**	**Shoulder pain (*****n** =* **133)**	**No shoulder pain (*****n** =* **106)**	***P-*** **value**	**Total (*****n** =* **239)**	**Shoulder pain (*****n** =* **142)**	**No shoulder pain (*****n** =* **97)**	***P-*** **value**	**Total (*****n** =* **236)**	**Shoulder pain (*****n** =* **136)**	**No shoulder pain (*****n** =* **100)**	***P-*** **value**
Age, (y) (*x* ± SD)	70 ± 9 (50, 94)	71 ± 9 (52,94)	70 ± 9 (50,91)	0.564	70 ± 9 (50,94)	71 ± 9 (52,94)	70 ± 9 (50,91)	0.418	70 ± 9 (50,94)	71 ± 9 (52,94)	70 ± 9 (50,91)	0.495
Male, *n* (%)	148 (62%)	75 (56%)	73 (69%)		148 (62%)	80 (56%)	68 (70%)		146 (62%)	75 (55%)	71 (71%)	
Female, *n* (%)	91 (38%)	58 (44%)	33 (31%)	0.048^*^	91 (38%)	62 (44%)	29 (30%)	0.031^*^	90 (38%)	61 (45%)	29 (29%)	0.013^*^
**Past medical history**												
Hypertention, *n* (%)	201 (84%)	111 (84%)	90 (85%)	0.761	201 (84%)	118 (83%)	83 (86%)	0.608	199 (84%)	114 (84%)	85 (85%)	0.806
Atrial fibrillation, *n* (%)	31 (13%)	22 (17%)	9 (9%)	0.066	31 (13%)	23 (16%)	8 (8%)	0.072	30 (13%)	21 (15%)	9 (9%)	0.142
**Main type stroke**												
Infarction, *n* (%)	197 (82%)	106 (80%)	91 (86%)	0.241	197 (82%)	115 (81%)	82 (85%)	0.409	195 (83%)	110 (81%)	85 (85%)	0.388
Cerebral hemorrhage, *n* (%)	39 (16%)	26 (20%)	13 (12%)		39 (16%)	26 (18%)	13 (13%)		38 (16%)	25 (18%)	13 (13%)	
Subarachnoid hemorrhage, *n* (%)	3 (1%)	1 (1%)	2 (2%)		3 (1%)	1 (1%)	2 (2%)		3 (1%)	1 (1%)	2 (2%)	
**Hemiplegic side**												
Left, *n* (%)	115 (48%)	66 (50%)	49 (46%)	0.601	115 (48%)	68 (48%)	47 (49%)	0.931	113 (48%)	64 (47%)	49 (49%)	0.768
Right, *n* (%)	121 (51%)	65 (49%)	56 (53%)	0.543	121 (51%)	72 (51%)	49 (51%)	0.977	120 (51%)	70 (52%)	50 (50%)	0.823
Both, *n* (%)	3 (1%)	2 (2%)	1 (1%)	1.000	3 (1%)	2 (1%)	1 (1%)	1.000	3 (1%)	2 (2%)	1 (1%)	1.000
**Stroke location**												
Massive, *n* (%)	27 (11%)	25 (19%)	2 (2%)	<0.001^**^	27 (11%)	26 (18%)	1 (1%)	<0.001^**^	27 (11%)	26 (19%)	1 (1%)	<0.001^**^
Cortical, *n* (%)	38 (16%)	21 (16%)	17 (16%)	0.958	38 (16%)	23 (16%)	15 (16%)	0.879	38 (16%)	22 (16%)	16 (16%)	0.971
Brain stem, *n* (%)	34 (14%)	18 (14%)	16 (15%)	0.732	34 (14%)	17 (12%)	17 (18%)	0.227	33 (14%)	16 (12%)	17 (17%)	0.252
Pontine, *n* (%)	20 (8%)	13 (10%)	7 (7%)	0.379	20 (8%)	13 (9%)	7 (7%)	0.595	19 (8%)	12 (9%)	7 (7%)	0.611
Basal gangregion, *n* (%)	35 (15%)	20 (15%)	15 (14%)	0.847	35 (15%)	21 (15%)	14 (14%)	0.939	34 (14%)	20 (15%)	14 (14%)	0.879
Thalamic, *n* (%)	9 (4%)	8 (6%)	1 (1%)	0.088	9 (4%)	8 (6%)	1 (1%)	0.136	9 (4%)	8 (6%)	1 (1%)	0.112
Undefined, *n* (%)	8 (3%)	4 (3%)	4 (4%)									

% indicates proportion of the total group in each column.

^**^P < 0.01, significant differences between the groups with and without shoulder pain; ^*^P < 0.05, significant differences between the groups with and without shoulder pain.

By comparing the baseline clinical factors between the two groups, we found that female patients (χ^2^ = 3.895, *P* < 0.05), patients with large-area infarction (χ^2^ = 16.833, *P* < 0.001) were more prone to develop shoulder pain at initial assessment. However, there was no significant correlation between shoulder pain and age, comorbidity of hypertension, comorbidity of atrial fibrillation, stroke type, hemiplegic side, cortical infarction, brainstem infarction, basal ganglia infarction and pontine infarction.

At follow-up I and II, we compared the differences of basic clinical factors between patients with shoulder pain and patients without shoulder pain again, and found the same results.

### Relationship between HSP and muscle tone, subluxation, shoulder PROM and ADL

We found that a greater proportion of patients in the group with shoulder pain (53%) had biceps brachii or triceps brachii muscle tone dystonia than those without shoulder pain (23%) after stroke, and shoulder pain was significantly associated with dystonia (χ^2^ = 17.891, *P* < 0.001), especially increased biceps / triceps muscle tension. However, we didn't find an association between shoulder pain and decreased biceps/triceps muscle tension (χ^2^ = 2.111, *P* = 0.146).

Shoulder pain was also significantly associated with subluxation and restricted shoulder PROM. Gleno-humeral subluxation occurred in 34% (45/133) of patients with shoulder pain, which was significantly higher than that in patients without shoulder pain (χ^2^ = 41.058, *P* < 0.001). We found a relationship between shoulder pain and restricted shoulder PROM in terms of mild, moderate and severe incidence (χ^2^ = 106.352, *P* < 0.001; χ^2^ = 22.744, *P* < 0.001; χ^2^ = 57.755, *P* < 0.001) (Table 2).

**Table 2:** Clinical examinations and their relation to hemiplegic shoulder pain.

	**Shoulder pain (*n =* 133)**	**No shoulder pain (*n =* 106)**	***P-*value**
**Dystonia**^a^, ***n*** **(%)**	71 (53%)	24 (23%)	<0.001^*^
Increased	50 (38%)	14 (13%)	<0.001^*^
Decreased	21 (16%)	10 (9%)	0.146
**Subluxation (** ***n*** **, %)**	45 (34%)	1 (1%)	<0.001^*^
**Shoulder PROM** ^b^			
Mild restriction hspace^*^0pt (*n*, %)	42 (32%)	103 (97%)	<0.001^*^
Moderate restriction hspace^*^0pt (*n*, %)	31 (23%)	2 (2%)	<0.001^*^
Severe restriction hspace^*^0pt (*n*, %)	58 (44%)	1 (1%)	<0.001^*^

^a^Dystonia: the muscle tension of biceps or triceps on the hemiplegic side.

^b^Shoulder PROM: Mild restriction: flexion/abduction 140°-180°, or internal/external rotation 60°-90°; Moderate restriction: flexion/abduction 90°-140°, or internal/external rotation 45°-60°; Severe restriction: flexion/abduction 0°-90°, or internal/external rotation 0°-45°.

^*^Statistically significant.

Patients without shoulder pain were more independent than those with shoulder pain, including dressing (*t* = −5.418, *P* < 0.001), grooming (*t* = −3.306, *P* = 0.001 < 0.01), feeding (*t* = −2.327, *P* = 0.021 < 0.05) and bathing (*t* = −2.781, *P* = 0.006 < 0.01) (Table 3).

**Table 3:** ADL of upper limbs and their relation to hemiplegic shoulder pain.

	**Shoulder pain (*n =* 133)**	**No shoulder pain (*n =* 106)**	***P*-value**
MBI (upper limbs) ($\bar{x}$ ± SD)	10.2 ± 7.9	14.6 ± 8.6	<0.001^*^
Eating ($\bar{x}$ ± SD)	4.8 ± 3.8	6.0 ± 3.9	0.021^*^
Shower ($\bar{x}$ ± SD)	0.8 ± 1.1	1.3 ± 1.5	<0.01^*^
Dressing ($\bar{x}$ ± SD)	2.9 ± 2.6	4.8 ± 3.1	<0.001^*^
Making up ($\bar{x}$ ± SD)	1.7 ± 1.6	2.4 ± 1.6	<0.01^*^

^*^Statistically significant.

### Relationship between HSP and Infection, immunity, Vitamin D3 and blood glucose level

We found that most stroke patients had elevated SAA and ESR, as well as vitamin D3 deficiency, regardless of whether they had shoulder pain. Vitamin D3 deficiency was present in 83% of stroke patients, with a mean value of 36.5 nmol/L vitamin D3 in serum, while 16% of stroke patients had severe deficiency. 61% of stroke patients had increased ESR, with a mean value of 29.6 mm/h in serum. 71% of stroke patients had increased SAA with a mean value of 34.9 mg/L. However, we did not find a significant correlation between shoulder pain and elevated SAA and ESR and vitamin D3 deficiency. There was also no significant difference in fasting blood glucose, 2 h postprandial blood glucose and glycosylated hemoglobin between patients with shoulder pain and those without shoulder pain (Table 4).

**Table 4:** Infection, immunity, vitamin D3 and blood glucose level and their relation to hemiplegic shoulder pain.

	***N* (SP/NSP)**	***N* = (x̄ ±SD)**	**Shoulder pain**	**No shoulder pain**	***P*-value**
WBC (10*9/L)	239 (133/106)	6.4 ± 1.8	6.3 ± 1.7	6.6 ± 1.8	0.222
NEUT (%)	239 (133/106)	61.7 ± 8.9	60.8 ± 8.9	62.7 ± 8.7	0.104
CRP (mg/L)	239 (133/106)	6.9 ± 7.5	6.5 ± 6.2	7.5 ± 8.9	0.312
ESR (mm/h)	196 (108/88)	29.6 ± 22.7	30.8 ± 21.3	28.1 ± 24.2	0.417
SAA (mg/L)	131 (69/62)	34.9 ± 22.7	36.3 ± 61.8	33.3 ± 42.2	0.747
VitD3 (nmol/L)	233 (128/105)	36.5 ± 17.5	35.7 ± 18.0	37.5 ± 16.9	0.424
HbA1c (%)	127 (63/64)	7.0 ± 1.3	6.9 ± 1.4	7.1 ± 1.3	0.283
FPG (mmol/L)	196 (105/91)	5.9 ± 1.6	5.9 ± 1.7	5.9 ± 1.5	0.954
P2hPG (mmol/L)	116 (58/58)	11.1 ± 3.2	10.7 ± 3.4	11.6 ± 3.0	0.126
		*n* (%)			
CRP↑ (yes)		58 (24.3%)	29 (22%)	29 (27%)	0.320
ESR↑ (yes)		119 (60.7%)	70 (65%)	49 (56%)	0.193
SAA↑ (yes)		93 (71.0%)	51 (74%)	42 (68%)	0.437
VitD3 deficiency^a^ (yes)		194 (83%)	108 (84%)	86 (82%)	0.615
Mild		45 (23.2%)	23 (21%)	22 (26%)	0.482
Moderate		119 (61.3%)	66 (61%)	53 (62%)	0.941
Severe		30 (15.5%)	19 (18%)	11 (13%)	0.358

A, vitamin D3 deficiency: mild: VitD3 (40, 50); moderate: VitD3 (20, 40); severe: VitD3 <20

SP, shoulder pain; NSP, no shoulder pain.

### Ultrasonographic manifestations in patients with HSP

Seventy-seven patients with shoulder pain underwent shoulder ultrasound examination finally. We found that 69% (53/77) of HSP patients developed supraspinatus tendinitis, 66% (51/77) of HSP patients developed tendinitis of the long head of biceps brachii and 24% (18/77) of HSP patients suffered from SA-SD bursitis (Table 5). Tendon thickness of more than 8 mm is generally considered diagnostic of tendon disease (36), and hypoechoic areas >2 mm around the tendon of the long head of biceps brachii are considered diagnostic of tendinitis of the long head of biceps brachii (37), whereas the diagnostic criterion for SA-SD bursitis is a cumulative fluid thickness of more than 2 mm (38). In our study, the average thickness of supraspinatus tendon was 8.1 ± 1.4 mm on the hemiplegic side and was 6.8 ± 1.1 mm on the unaffected side in patients with HSP. The difference between the two groups had statistical significance (*t* = 6.197, *P* < 0.001) (Table 6).

**Table 5:** Ultrasonic findings of shoulder joint in patients with HSP.

	***N* = 77, n (n%)**	**Mean tendon thickness (mm)**
**Supraspinatus tendon**		
Normal	21 (27%)	6.4 ± 0.7
Tear	3 (4%)	-
Tendinitis in hemiplegic side	40 (52%)	9.0 ± 0.9
Tendinitis in healthy side	3 (4%)	8.5 ± 0.5
Tendinitis bilateral	10 (13%)	
Hemiplegic side		9.0 ± 0.8
Healthy side		8.9 ± 0.6
		Mean depth of effusion (mm)
**Tendon of biceps**		
Normal	26 (34%)	-
Effusion in hemiplegic side	26 (34%)	3.0 ± 0.7
Effusion in healthy side	7 (9%)	2.6 ± 0.5
Effusion bilateral	18 (23%)	
Hemiplegic side		3.0 ± 0.7
Healthy side		2.8 ± 0.6
**SA-SD bursae**		
Normal	59 (76%)	-
Effusion in hemiplegic side	16 (21%)	3.2 ± 1.6
Effusion in healthy side	2 (3%)	3.2 ± 0.3
Effusion bilateral	0 (0%)	

SA-SD, subacrominal-subdeltoid.

**Table 6:** The thickness of supraspinatus tendon on bilateral sides in HSP patients.

	**Hemiplegic side**	**Healthy side**	***P-*value**
Thickness of supraspinatus tendon (*x* ± SD)	8.1 ± 1.4	6.8 ± 1.1	<0.001^*^

^*^Statistically significant.

### HSP affected rehospitalization rates and the return to home

**Figure 4:**
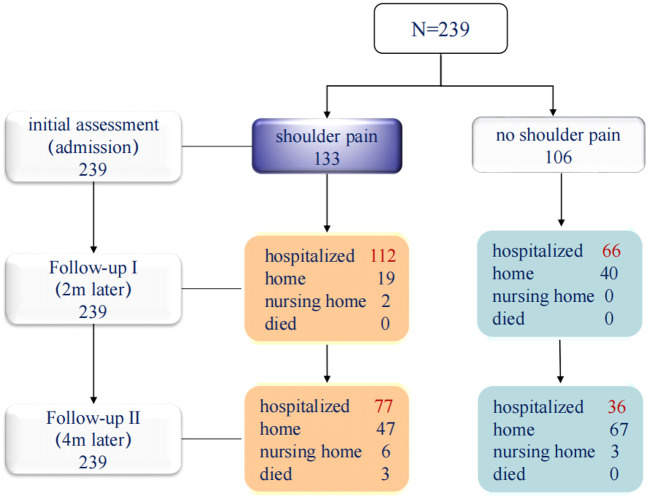
Rehabilitation course at follow-ups.

We found that the rehospitalization rate of patients with HSP was 84% (112/133) at 2 month of follow-up I, and 59% (77/130) at 4 month of follow-up II, compared with 62% (66/106) and 34% (36/106) of patients without shoulder pain, respectively. Comparing to patients with HSP, patients without shoulder pain were more likely to return to their families. 38% (40/106) of patients without shoulder pain returned to home at 2 months of follow-up, compared with only 14% (19/133) of patients with shoulder pain. At 4 months of follow-up, the proportions of patients without shoulder pain and those with shoulder pain who returned home were 62% (67/106) and 35% (47/133), respectively (Figure 4).

## Discussion

### Main findings

In this study, we tried to observe the prevalence of post-stroke HSP in the middle-aged and elderly population, find out the risk factors associated with HSP, and investigated the effect of HSP on stroke outcomes. We found that 56% of stroke patients admitted to the rehabilitation ward suffered from HSP. We observed that HSP last for a long time once it developed. The prevalence of HSP was 59% at 2 months follow-up and 55% at 4 months follow-up. Female, large-area stroke, elevated biceps/triceps tone, subluxation and limited shoulder movement are the risk factors for HSP. Age, history of hypertension, history of atrial fibrillation, stroke type, hemiplegic side, cerebral cortex stroke, brain stem stroke, basal ganglia stroke, thalamic stroke, blood glucose level, inflammatory markers, and vitamin D3 were not significantly associated with HSP. Shoulder ultrasonography showed that 69% of patients with HSP had supraspinatus tendinitis, 66%had long head of biceps brachii, and 23% had SA-SD bursitis. We also found that the thickness of supraspinatus tendon on the hemiplegic side was significantly higher than that on the unaffected side in patients with HSP. Besides, patients with HSP had significantly lower self-care ability and significantly longer hospital stay, comparing with patients without shoulder pain.

### Prevalence

Due to medical level, stroke duration, study design and other factors, different studies have obtained prevalence of HSP. Previous studies (4, 5, 29, 39) have found that the prevalence of HSP ranges between 20 and 60%, which is close to the results obtained in our study. Previous studies on HSP have mostly been performed in neurology or stroke unit, while our study was performed in rehabilitation wards. In this study, we demonstrated that the changes of HSP during the recovery period with the course of the disease, and paid more attention to the impact of shoulder pain on the recovery of limb function, which is conducive to guiding rehabilitation strategies.

### Risk factors of HSP

Our study found that female patients were more likely to develop HSP. However, previous studies showed that shoulder pain was not associated with gender. In one study (13), it was shown that there was no significant difference between patients with shoulder pain and patients without shoulder pain in terms of gender proportion at 1 week, 1 month and 6 months after stroke. The method of judging HSP in their study was mainly to ask the patients or their caregivers whether they ever had shoulder pain, without paying attention to shoulder pain during exercise. Whereas, in our study, in addition to the shoulder pain questionnaire, our study also performed a detailed shoulder physical examination and asked patients if they had shoulder pain during passive and active shoulder movement. From above, different methods of judging shoulder pain may lead to different results. In addition, we also found that shoulder pain occurs mostly during or after exercise, so attention should be paid to avoid causing shoulder joint injury in patients during rehabilitation training.

Ratnasabapathy et al. (13) found that left hemiplegia, that is, stroke in the right cerebral hemisphere, is a risk factor for hemiplegic shoulder pain. However, some studies believe that there is no significant correlation between hemiplegic shoulder pain and hemiplegic side. The clinical manifestations and dysfunction of different stroke location are different. However, the right hemisphere includes many lesion locations. Further research is needed to explore the relationship between shoulder pain and stroke location.

Our study is the first to observe the relationship between shoulder pain and different stroke location. By collecting the cranial imaging examination report of patients at the onset of stroke, and comparing the proportion of different stroke location between two groups, it is found that the incidence of shoulder pain in patients with large-area stroke is higher. Complications and seriously damaged motor function, which can lead to shoulder pain, usually occurs in patients with large-area stroke. Isaksson et al. (40) of Sweden found that the score of The National Institutes of Health Stroke Scale (NIHSS) in patients with shoulder pain was significantly higher in stroke patients with severe upper limb paralysis. Pong et al. (41) in Taiwan found that hemiplegic shoulder pain was associated with lower motor function levels in both acute and chronic stages of stroke. Nadler et al. (29) found that patients with shoulder abductor muscle strength ≤ grade 2 have a higher incidence of shoulder pain. However, different hospitals may have different imaging diagnostic criteria for large-area stroke, so quantitative indicators are needed. Patients with thalamic stroke are prone to thalamic pain. Thalamic pain is a part of central pain, with an incidence rate of 8–17% (42). Mild stimulation can lead to severe pain and various forms of sensory abnormalities. However, our study did not find the relationship between thalamic stroke and hemiplegic shoulder pain. The possible reason is that fewer people with simple thalamic stroke were included. Prefrontal lobe and anterior cingulate cortex are involved in pain adaptation. Stroke patients with frontal lobe injury have reduced pain adaptation, resulting in hyperalgesia and prone to shoulder pain (11).

Spasticity is closely related to hemiplegic shoulder pain. Our study found that patients with elevated biceps/triceps tone have a higher incidence of shoulder pain. Flexor spasm of upper limb after stroke is manifested in shoulder adduction and pronation (43). The tone of trapezius and rhomboid muscles increases, and the scapula is raised, which is prone to develop subacromial impingement syndrome (SIS), resulting in shoulder pain (44). Repeated friction and extrusion stimulate highly dense pain receptors in soft tissue. Many studies have observed that the proportion of spasm in patients with shoulder pain is higher than that in patients without shoulder pain. Poulin et al. (45) observed 94 patients with HSP for 2 years and found that patients with shoulder pain were more prone to limb spasm. Barlak et al. (39) found that 60% patients with HSP suffered from upper limb spasm.

A mount of studies found that some treatments, which can alleviate muscle spasm, such as botulinum toxin injection, shock wave therapy and surgical therapy, can significantly cure spasm and shoulder pain. A systematic review (46) suggested that botulinum toxin injection significantly reduced the VAS score of shoulder pain compared with steroid or placebo injection. However, the injection site and dose of botulinum toxin are still quite different in different studies. For patients with HSP and spasm, Yelnik et al. (47) injected 500 units of botulinum toxin or placebo into the subscapular muscle, Lim et al. (48) injected 100u botulinum toxin or normal saline into infraspinatus muscle, pectoralis major muscle and subscapularis muscle, Bhakta et al. (49) injected 400–1,000 units of gemcitabine botulinum toxin or 100–200 units of botulinum toxin into biceps brachii, flexor digitorum and flexor carpi, Marco et al. (50) injected 500 units of botulinum toxin into the pectoralis major muscle on the hemiplegic side, finally their results showed that botulinum toxin treatment could effectively improve shoulder pain.

We found that patients with subluxation are more prone to develop shoulder pain. However, the relationship between glenohumeral subluxation and hemiplegic shoulder pain is controversial. After stroke, the muscles around the shoulder are weak and can't effectively resist gravity. There is an obvious gap between the acromion and the humeral head, which is called glenohumeral joint subluxation. The change of the position of the humeral head makes the soft tissue around the shoulder easy to be damaged (51), resulting in shoulder pain. A systematic review (7) to assess the relationship between subluxation and shoulder pain enrolled 14 studies, of which 7 studies found a close relationship between hemiplegic shoulder pain and subluxation, and 7 studies found no significant correlation between subluxation and hemiplegic shoulder pain. Through palpation, the judgment of subluxation is greatly affected by the patient's body position and the subjective factors of the evaluator. The acromion-greater tuberosity distance (AGTD) under ultrasonography is a quantitative index to measure the subluxation. Korkmaz et al. (52) found that the AGTD is strongly related to biceps tendinitis, which can lead to shoulder pain. Many studies have pointed out that transcutaneous nerve electrical stimulation (TENS) around shoulder (53), wearing of shoulder orthosis (54) and kinesiology taping (18) can significantly improve shoulder subluxation and shoulder pain.

From the perspective of molecular, our study first observed the relationship between HSP and serological indicators such as blood glucose level, vitamin D3, ESR and infection indexes. It was found that there was no significant difference in fasting blood glucose, 2 h postprandial blood glucose, glycosylated hemoglobin, vitamin D3, ESR and infective situation between patients with shoulder pain and patients without shoulder pain.

Diabetes is related to a variety of musculoskeletal diseases. Hao et al. (55) found that diabetes is the independent risk factor of HSP. Roosink et al. (56) in the Netherlands also found that patients with HSP had a higher proportion of diabetes. Hadianfard et al. (28) of Iran used blood glucose value to judge whether the diagnosis of diabetes was reasonable when observing the relationship between HSP and diabetes, and found that there was a significant relationship between diabetes and HSP. Shoulder pain may be related to the course of diabetes, its complications and other factors, so the correlation between HSP and blood glucose remains to be further studied.

Vitamin D3 deficiency is considered to be associated with chronic pain (57). When the concentration of serum vitamin D3 is lower than 50 nmol/L, it is unfavorable to bone health (58). Vitamin D deficiency stimulates the increase of parathyroid secretion, resulting in secondary hyperparathyroidism. Parathyroid hormone can activate osteoblasts and stimulate osteoblasts to transform into mature osteoclasts. Osteoclasts dissolve the collagen matrix in bone, resulting in osteoporosis (59) and painful osteomalacia (60). A rat study (61) found that pills of vitamin D combined with raloxifene can effectively enhance the connection between rotator cuff tendon and bone, so as to promote the function of rotator cuff. A study (35) have found that pain has been significantly relieved after 16 weeks of vitamin D supplementation, however, some studies believe that there is a lack of sufficient evidence to prove that vitamin D treatment is beneficial to chronic pain (62). Vitamin D3 deficiency is common in stroke patients, however, our study found that there is no significant correlation between shoulder pain and vitamin D3. One of the factors that can't be ignored is that most patients are taking vitamin D3 supplements such as calcium or rogate during hospitalization, which may mask the effect of vitamin D3 deficiency on pain. Further research is needed to explore the relationship between vitamin D3 and HSP.

The soft tissue about shoulder of stroke patients is prone to be injured, which can activate the repair mechanism of the human body and start the inflammatory response. At present, a newly prospecting bioactive therapy called platelet rich plasma therapy can promote the inflammatory response and activate a variety of growth factors. It is applied to patients after rotator cuff tear surgery to promote rotator cuff repair (63). So we guess that HSP may be related to infection and immune factors, however, we did not find any significant result. One of the reasons is that there are insufficient samples or relatively single infection and immune indexes. Further research is still needed to explore the relationship between infection and immune factors and HSP.

In addition, HSP may also be related to poor motor function, paresthesia, and CRPS in hemiplegic limb, which were not focus on in this study. Meta-analysis (9) suggested that severe upper limb motor dysfunction is considered an important risk factor for post-stroke shoulder pain in the first year after stroke. Gamble et al. (12) evaluated light touch sensation, needle sensation and temperature sensation on the face, shoulder, forearm, hand, calf and foot of stroke patients, and found that patients with shoulder pain had a higher proportion of paresthesia, and pain was closely related to paresthesia on the hemiplegic side. Because of increased sympathetic nerve activity, inflammation, endothelial dysfunction and immune system defects, 21–31% of patients (64) develop CRSP after stroke and they typically suffered from shoulder pain. From above, the influencing factors of shoulder pain after hemiplegia are very complex and require in-depth study.

### The value of shoulder ultrasonography in the diagnosis of HSP

Shoulder joint ultrasonography provides an objective basis for the etiological diagnosis of shoulder pain. In our study, GE logiqe9 instrument was used to perform shoulder joint ultrasonography for patients with shoulder pain. The results showed that 69% of patients had supraspinatus tendinitis and 66% of patients had tendinitis of the long head of biceps brachii, which was the most common lesion in patients with hemiplegic shoulder pain. The thickness of supraspinatus tendon of hemiplegic side in patients with shoulder pain was significantly higher than that of healthy side. This is the first study of quantitative analysis of shoulder lesions in patients with hemiplegic shoulder pain using shoulder ultrasonography findings.

Different studies have different research samples, inspection instruments and observation indicators, therefore the conclusions are different. Lin PH (65) retrospectively analyzed the shoulder joint ultrasound results of 26 patients with hemiplegic shoulder pain. It was found that 73.08% of patients had effusion in the SA-SD bursae, 69.23% of patients had effusion in the tendon sheath of the long head of the biceps brachii, and the most common lesion of hemiplegic shoulder pain was the SA-SD bursitis. Korkmaz et al. (52) pointed out that SA-SD bursitis is a risk factor for hemiplegic shoulder pain. Supraspinatus tendon disease is a predictor of shoulder pain within 6 months after stroke (66). Falsetti et al. (67) observed that the incidence of supraspinatus tendon disease in stroke patients was 42.2%.

### Shoulder pain affect ADL

We found that shoulder pain reduced quality of life, which may hinder the improvement upper limb motor function in stroke patients. Adey-Wakeling et al. (68) measured quality of life through EQ-5D-3L, a visual analog scale and a 5-dimension descriptive system (dimensions include mobility, self-care, usual activities, pain/discomfort, and anxiety/depression), and also found that HSP reduces quality of life after stroke. Chae et al. (69) measured post-stroke shoulder pain with the Brief Pain Inventory question 12, a self-reported 11-point numeric rating scale (NRS) that assesses “worst pain” in the last 7 days, and found that post-stroke shoulder pain was significantly associated with reduced quality of life.

## Innovation

This is the first study to use shoulder ultrasonic findings to make quantitative analysis of shoulder joint lesions in patients with hemiplegic shoulder pain. There is no objective quantitative data in previous studies. Shoulder joint ultrasound is of great significance in the etiological diagnosis of hemiplegic shoulder pain. From multi-dimensional aspects such as molecular, structural and functional, we observed comprehensive risk factors of hemiplegic shoulder pain. Involving some objective data, the information is more reliable, which is a relatively novel place of this study.

From the perspective of rehabilitation, our study observed the evolution of the incidence of shoulder pain over time after systematic rehabilitation treatment. This is the first cross sectional study of hemiplegic shoulder pain in a specific age population and followed up after rehabilitation treatment. The age range of previous studies was large, ranging from about 20 to more than 90 years old. And many indicators observed in the study are elderly-age-related. Our study observed the middle-aged and elderly people, all over the age of 50, which excluded the interference of risk factors of young stroke.

## Limitations

However, our study has some limitations. First, most of our data were collected from rehabilitation scales and lacked sufficient quantitative and objective measures, which may introduce some bias to evaluation. Second, we defined post-stroke shoulder pain as occurring and persisting for lasting more than 48 h. At present, there is a lack of uniform criteria for the definition of HSP. In fact, the degree of shoulder pain is one of the important factors we should consider. This may affect the grouping of patients with shoulder pain. Third, this study is a single-center study with a limited sample size, and the applicability and generalizability of the findings will be limited. Finally, ultrasonography examination was performed only in patients with shoulder pain, and it is difficult to find a direct relationship between imaging and clinical impairments. Tendinitis and bursa effusion seen in this study may be associated with older age rather than stroke. In future studies, we will apply objective measures and increase interventions, and increase sample size to carry out multicenter clinical trials to identify risk factors for HSP. At the same time, we will compare images from stroke patients with shoulder pain, stroke patients without shoulder pain, and healthy controls to find a direct relationship between images and injuries.

## Conclusions

Our study found that hemiplegic shoulder pain has a high incidence and can last for several months. Female, large-scale stroke, elevated muscle tone, subluxation and limited shoulder movement may be important risk factors for hemiplegic shoulder pain. Shoulder joint ultrasound has certain value in the diagnosis of HSP. In addition, HSP increases the readmission rate of patients to some extent. Pain relief may better promote the recovery of upper limb motor function and shorten the in-hospital rehabilitation time.

## Data availability statement

The original contributions presented in the study are included in the article/Supplementary material, further inquiries can be directed to the corresponding authors.

## Ethics statement

The studies involving human participants were reviewed and approved by Ethics Committee of Ruijin Hospital, Shanghai Jiao Tong University School of Medicine. The patients/participants provided their written informed consent to participate in this study.

## Author contributions

JW and QX: conception and design of the study and data confirmation. YL: data collection, analysis of data, statistical analysis, and drafting the manuscript. SY: data collection and analysis. LC, YB, LG, and HP: acquisition of data. All authors contributed to the article and approved the submitted version.
