# Supplementary material for: Prevalence, risk factor and outcome in middle-aged and elderly population affected by hemiplegic shoulder pain: An observational study
Source: Front Neurol. 2023 Jan 12;13:1041263. doi: 10.3389/fneur.2022.1041263 (PMC9879055; doi:10.3389/fneur.2022.1041263)
Supplement: Supplementary file 1 [file Table_1.docx]

**shoulder pain questionnaire and relevant clinical physical examination**

**shoulder pain questionnaire**

| **Questions** | **Initial/follow-up I/follow-up II** |
| --- | --- |
| 1.Have you ever suffered from shoulder pain?  A. yes B. no | / / |
| 2.Does your shoulder pain occur after stroke? (if no, go to Q3,or go to Q4)  A. yes B. no | / / |
| 3.Does it get worse after stroke?  A. yes B. no | / / |
| 4.When does it start after stroke?  A. 2 weeks B.1 months C.2months D. 3months E. others | / / |
| 5.Does it serious?  A. VAS score 1-3 B. VAS score 4-6 C. VAS score 7-10 | / / |
| 6. How often does it hurt?  A. Daily B. every two days C. 2h/day D. 4h/day | / / |
| 7. Whether it occurs after the movement?  A. after the movement B. occurs at rest | / / |
| 8. wake up at night because of shoulder pain?  A. yes B. no | / / |
| 9.which side of shoulder pains?  A. hemiplegic side B. healthy side C. both sides | / / |
| 10. Are you taking painkillers? (if no, pass Q11)  A. yes B. no | / / |
| 11.What painkillers are you take?  A. Celebrex B. Acetaminophen C. Meloxicam D. Tramadol E. Ointment F. others | / / |
| 12. Are you taking drugs for reducing muscle tone?  A. yes B. no | / / |
| 13. Have you ever had physical therapy on your painful shoulder?  A. laser B. microwave C. TENS* D. ultrasonic E. infrared F. Extracorporeal shock wave G. no H. others | / / |

*TENS: Transcutaneous electrical nerve stimulation

**clinical physical examination**

|  | **Initial/follow-up I/follow-up II** |
| --- | --- |
| 1.Muscular tension of biceps or triceps on the hemiplegic side? (if B, go to 2, or go to 3)  A. normal B. increased C. decreased | / / |
| 2.Ashworth: A.0 B.1 C.1+ D.2 E.3 F.4 | / / |
| 3.subluxation (palpation under seat position)  A. yes B. no | / / |
| 4.shoulder positive range of motion:  anteflexion  retroflexion  abduction  internal rotation  external rotation | / / |
| 5. ADL* of upper limbs:  feeding  bathing  dressing  grooming | / / |
| 6. shoulder tenderness point:  A. Subcorostral process B. Long head tendon of biceps brachii C. Subacromial D. Attachment point of teres minor muscle | / / |
| 7. Swelling of hemiplegic hand  A. yes B. no | / / |

ADL: activity of daily living
